# Supplementary material for: Indications, Detection, Completion and Retention Rates of Capsule Endoscopy in Two Decades of Use: A Systematic Review and Meta-Analysis
Source: Diagnostics (Basel). 2022 Apr 28;12(5):1105. doi: 10.3390/diagnostics12051105 (PMC9139699; doi:10.3390/diagnostics12051105)
Supplement: Supplementary file 1 [file diagnostics-12-01105-s001.zip › supplementary figures ALL.pdf]

diagnostics

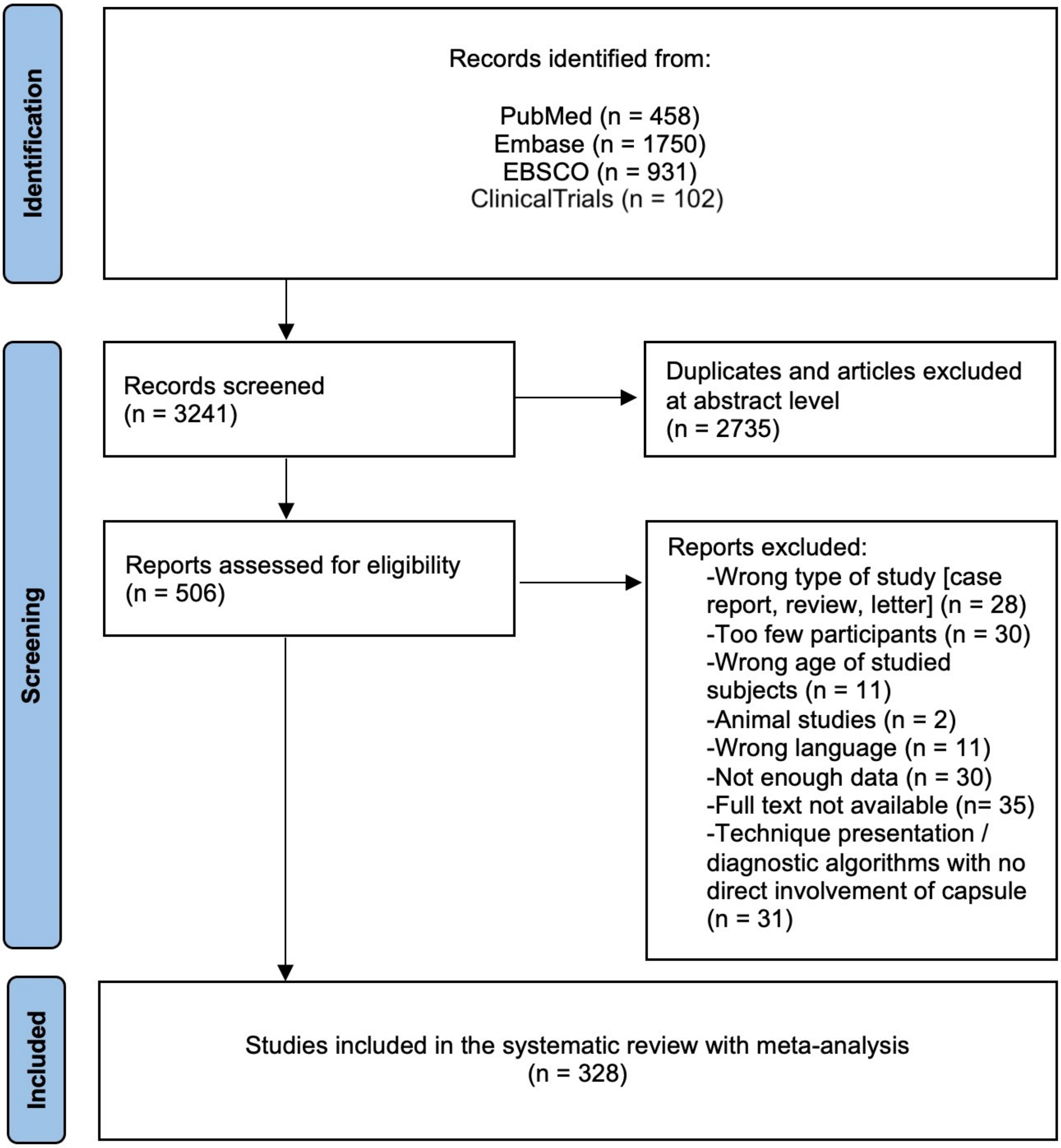

Figure S1. Study's consort flow diagram.

Detection rate in CD by capsule type

Model	Capsule type	Study name	Event rate	Lower limit	Upper limit	Z-Value	p-Value
	CCE	Shi et al., 2017	0,760	0,685	0,822	6,029	0,000
	CCE	D'Haens et al., 2015	0,875	0,733	0,947	4,070	0,000
Fixed	CCE		0,779	0,714	0,833	7,110	0,000
Random	CCE		0,816	0,597	0,930	2,662	0,008
	Combi	Tomer Greener et al., 2016	0,518	0,389	0,645	0,267	0,789
Fixed	Combi		0,518	0,389	0,645	0,267	0,789
Random	Combi		0,518	0,195	0,827	0,094	0,925
	PCC	Tai et al., 2020	0,548	0,447	0,646	0,932	0,351
	PCC	Tontini et al., 2020	0,561	0,408	0,703	0,779	0,436
	PCC	Leighton et al., 2017	0,733	0,622	0,821	3,874	0,000
	PCC	Eliakim et al., 2018	0,983	0,783	0,999	2,859	0,004
Fixed	PCC		0,621	0,552	0,685	3,423	0,001
Random	PCC		0,676	0,477	0,827	1,741	0,082
	SB	Papadopoulos et al., 2008	0,538	0,282	0,776	0,277	0,782
	SB	Petruzziello et al., 2010	0,938	0,782	0,984	3,708	0,000
	SB	Rodrigues-Pinto et al., 2016	0,789	0,726	0,842	7,428	0,000
	SB	Rondonotti et al., 2010	0,497	0,444	0,550	-0,109	0,913
	SB	Rosa et al., 2012	0,411	0,290	0,543	-1,329	0,184
	SB	Santos-Antunes et al., 2015	0,519	0,424	0,612	0,388	0,698
	SB	Tontini et al., 2017	0,300	0,164	0,483	-2,127	0,033
	SB	Tsibouris et al., 2013	0,995	0,927	1,000	3,755	0,000
	SB	Voderholzer et al., 2005	0,756	0,603	0,863	3,111	0,002
	SB	Wiarda et al., 2012	0,280	0,140	0,482	-2,120	0,034
	SB	Xavier et al., 2018	0,732	0,618	0,822	3,756	0,000
	SB	Xavier et al., 2018	0,763	0,690	0,823	6,206	0,000
	SB	Jensen et al., 2011	0,276	0,192	0,379	-4,023	0,000
	SB	Kopylov et al., 2015	0,846	0,721	0,921	4,435	0,000
	SB	Lee et al., 2019	0,808	0,613	0,918	2,884	0,004
	SB	Leighton et al., 2014	0,138	0,078	0,231	-5,656	0,000
	SB	Long et al., 2011	0,779	0,679	0,854	4,849	0,000
	SB	Marmo et al., 2005	0,710	0,530	0,841	2,259	0,024
	SB	Nemeth et al., 2016a	0,574	0,525	0,621	2,967	0,003
	SB	Niv et al., 2012	0,574	0,440	0,698	1,085	0,278
	SB	Alkhormi et al., 2018	0,250	0,034	0,762	-0,951	0,341
	SB	Bourreille et al., 2006	0,656	0,479	0,798	1,737	0,082
	SB	Carter et al., 2018	0,380	0,257	0,520	-1,680	0,093
	SB	Chong et al., 2005	0,488	0,344	0,634	-0,152	0,879
	SB	De Bona et al., 2006	0,395	0,254	0,556	-1,288	0,198
	SB	Efthymiou et al., 2009	0,690	0,503	0,830	1,989	0,047
	SB	Endo et al., 2008	0,833	0,194	0,990	1,039	0,299
	SB	Ersoy et al., 2006	0,929	0,423	0,996	1,748	0,081
	SB	Esaki et al., 2014	0,477	0,404	0,551	-0,606	0,544
	SB	Figueiredo et al., 2010	0,474	0,367	0,585	-0,453	0,651
	SB	Fireman et al., 2004	0,524	0,318	0,721	0,218	0,827
	SB	Flamant et al., 2013	0,833	0,751	0,892	6,233	0,000
	SB	Flemming et al., 2018	0,923	0,609	0,989	2,387	0,017
	SB	Gölder et al., 2006	0,722	0,481	0,879	1,816	0,069
	SB	Halling et al., 2014	0,850	0,704	0,931	3,917	0,000
	SB	Han et al., 2018	0,651	0,543	0,745	2,700	0,007
Fixed	SB		0,580	0,559	0,600	7,577	0,000
Random	SB		0,616	0,550	0,678	3,424	0,001
Fixed	Overall		0,593	0,574	0,611	9,555	0,000
Random	Overall		0,661	0,529	0,772	2,380	0,017

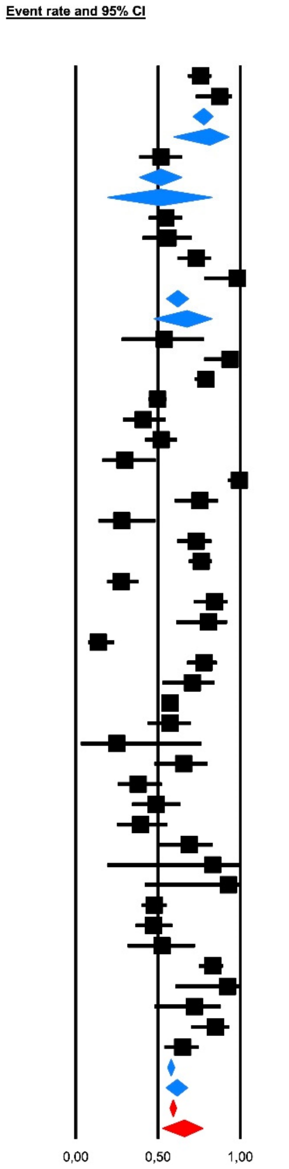

Figure S2. Detection rates in Crohn's disease by capsule type.

Completion rate in NL by capsule type

Model	Capsule type	Study name	Event rate	Lower limit	Upper limit	Z-Value	p-Value
	CCE	Parodi et al., 2018	0,997	0,957	1,000	4,146	0,000
	CCE	Pecere et al., 2020	0,802	0,744	0,849	8,301	0,000
	CCE	Rex et al., 2015	0,983	0,970	0,990	13,848	0,000
	CCE	Rondonotti et al., 2014	0,900	0,781	0,958	4,661	0,000
	CCE	Sacher Huvelin et al., 2010	0,913	0,886	0,934	15,425	0,000
	CCE	Saito et al., 2015	0,879	0,776	0,938	5,253	0,000
	CCE	Spada et al., 2011	0,881	0,805	0,929	6,765	0,000
	CCE	Voska et al., 2019	0,893	0,846	0,927	9,841	0,000
	CCE	Igawa et al., 2017	0,984	0,789	0,999	2,883	0,004
	CCE	Kroijer et al., 2018	0,661	0,589	0,726	4,244	0,000
	CCE	González-Suárez et al., 2020	0,986	0,947	0,997	5,996	0,000
Fixed	CCE		0,865	0,847	0,882	24,793	0,000
Random	CCE		0,921	0,860	0,957	7,467	0,000
	PC	Nakamura et al., 2021	0,496	0,437	0,555	-0,121	0,904
Fixed	PC		0,496	0,437	0,555	-0,121	0,904
Random	PC		0,496	0,127	0,870	-0,015	0,988
	SB	Rondonotti et al., 2008	0,694	0,607	0,768	4,193	0,000
	SB	Schulmann et al., 2005	0,850	0,704	0,931	3,917	0,000
	SB	Van Tuyl et al., 2006	0,556	0,251	0,823	0,333	0,739
	SB	Westerhof et al., 2009	0,700	0,473	0,859	1,736	0,082
	SB	Höög et al., 2012	0,441	0,354	0,531	-1,286	0,199
	SB	Haanstra, et al., 2017	0,865	0,801	0,910	7,897	0,000
Fixed	SB		0,672	0,624	0,717	6,627	0,000
Random	SB		0,707	0,512	0,848	2,073	0,038
Fixed	Overall		0,765	0,745	0,784	21,462	0,000
Random	Overall		0,782	0,455	0,939	1,718	0,086

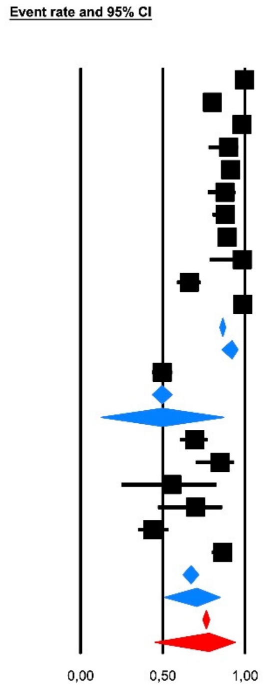

Figure S3. Completion rates in neoplastic lesions by capsule type.

Retention rate in NL by capsule type

Model	Capsule type	Study name	Event rate	Lower limit	Upper limit	Z-Value	p-Value
	CCE	Parodi et al., 2018	0,003	0,000	0,043	-4,146	0,000
	CCE	Pecere et al., 2020	0,002	0,000	0,035	-4,307	0,000
	CCE	Rex et al., 2015	0,001	0,000	0,011	-5,110	0,000
	CCE	Rondonotti et al., 2014	0,010	0,001	0,138	-3,247	0,001
	CCE	Sacher Huvelin et al., 2010	0,001	0,000	0,015	-4,940	0,000
	CCE	Saito et al., 2015	0,007	0,000	0,108	-3,445	0,001
	CCE	Spada et al., 2011	0,005	0,000	0,068	-3,802	0,000
	CCE	Toth et al., 2017	0,125	0,007	0,734	-1,287	0,198
	CCE	Van Gossum et al., 2009	0,002	0,000	0,024	-4,566	0,000
	CCE	Voska et al., 2019	0,002	0,000	0,034	-4,317	0,000
	CCE	Kroijer et al., 2018	0,003	0,000	0,043	-4,158	0,000
Fixed	CCE		0,004	0,002	0,008	-13,114	0,000
Random	CCE		0,004	0,001	0,009	-12,082	0,000
	Combi	Kim et al., 2020	0,006	0,001	0,041	-5,115	0,000
Fixed	Combi		0,006	0,001	0,041	-5,115	0,000
Random	Combi		0,006	0,001	0,055	-4,395	0,000
	MCCE	Wang et al., 2018a	0,011	0,001	0,154	-3,156	0,002
Fixed	MCCE		0,011	0,001	0,154	-3,156	0,002
Random	MCCE		0,011	0,001	0,188	-2,910	0,004
	PC	Nakamura et al., 2021	0,002	0,000	0,028	-4,456	0,000
Fixed	PC		0,002	0,000	0,028	-4,456	0,000
Random	PC		0,002	0,000	0,036	-4,106	0,000
	SB	Rondonotti et al., 2008	0,097	0,056	0,163	-7,353	0,000
	SB	Rondonotti et al., 2010	0,005	0,001	0,036	-5,233	0,000
	SB	Schulmann et al., 2005	0,025	0,004	0,157	-3,617	0,000
	SB	Sturniolo et al., 2005	0,071	0,004	0,577	-1,748	0,081
	SB	Van Weyenberg et al., 2010	0,024	0,001	0,287	-2,594	0,009
	SB	Höög et al., 2012	0,034	0,013	0,087	-6,585	0,000
	SB	Lim et al., 2015	0,070	0,032	0,147	-6,120	0,000
	SB	Cheon et al., 2007	0,036	0,005	0,214	-3,236	0,001
	SB	Haanstra, et al., 2017	0,003	0,000	0,049	-4,052	0,000
Fixed	SB		0,057	0,039	0,082	-13,982	0,000
Random	SB		0,040	0,021	0,073	-9,637	0,000
Fixed	Overall		0,031	0,022	0,043	-19,500	0,000
Random	Overall		0,008	0,002	0,040	-5,823	0,000

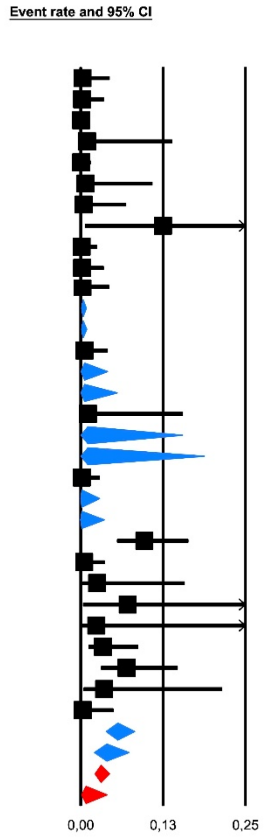

Figure S4. Retention rates in neoplastic lesions by capsule type.
